# Assessing Artificial Intelligence-Powered Responses to Common Patient Questions on Radiofrequency Ablation and Cryoanalgesia for Chronic Pain

**DOI:** 10.3390/jcm14196814

**Published:** 2025-09-26

**Authors:** Giuliano Lo Bianco, Marco Cascella, Silvia Natoli, Francesco Paolo D’Angelo, Emanuele Sinagra, Maurizio Marchesini, Emanuele Piraccini, Andrea Tinnirello, Felice Occhigrossi, Cesare Gregoretti, Christopher L. Robinson

**Affiliations:** 1 Department of Anaesthesia and Intensive Care, Fondazione Istituto “G. Giglio” Cefalù, 90015 Palermo, Italy; c.gregoretti@gmail.com; 2 Anesthesia and Pain Medicine, Department of Medicine, Surgery and Dentistry “Scuola Medica Salernitana”, University of Salerno, 84081 Baronissi, Italy; m.cascella@istitutotumori.na.it; 3 Department of Clinical-Surgical, Diagnostic and Pediatric Sciences, University of Pavia, 27100 Pavia, Italy; silvia.natoli@unipv.it; 4 Pain Unit, Fondazione IRCCS Policlinico San Matteo, 27100 Pavia, Italy; 5 Department of Anaesthesia, Intensive Care and Emergency, University Hospital Policlinico Paolo Giaccone, 90127 Palermo, Italy; francescods.dangelo@gmail.com; 6 Gastroenterology and Endoscopy Unit, Fondazione Istituto San Raffaele Giglio, 90015 Cefalù, Italy; emanuelesinagra83@googlemail.com; 7 Department of Anesthesia and Pain Medicine, Mater Olbia Hospital, 07026 Olbia, Italy; marchesinidoc@gmail.com; 8 Unit of Pain Management, Emergency Department, AUSL Bologna, 40033 Bologna, Italy; drpiraccini@gmail.com; 9 Anesthesia and Pain Management Unit, ASST Franciacorta, Iseo Hospital, 25049 Brescia, Italy; andrea.tinnirello@asst-franciacorta.it; 10 Pain Therapy Unit, San Giovanni-Addolorata Hospital, 00184 Rome, Italy; focchigrossi@hsangiovanni.roma.it; 11 Faculty of Medicine and Surgery, Saint Camillus International University of Health and Medical Sciences “UniCamillus”, 00131 Rome, Italy; 12 Division of Pain Medicine, Department of Anesthesiology and Critical Care, School of Medicine, Johns Hopkins University, Baltimore, MD 21205, USA; christopherrobinsonmdphd@outlook.com

**Keywords:** radiofrequency ablation, cryoanalgesia, neuromodulation, patient-entered care, patient education, artificial intelligence, ChatGPT, chronic pain management

## Abstract

**Background:** Radiofrequency ablation (RFA) and cryoanalgesia are minimally invasive procedures used when conservative medical management fails and there are positive diagnostic blocks. Although both radiofrequency ablation (RFA) and cryoanalgesia are straightforward techniques, the increasing workload of physicians is leading to less time available for patient interaction, including addressing questions about indications, feasibility, long-term effectiveness, and potential complications. Generative artificial intelligence (AI) chatbots, such as ChatGPT, have the potential to reduce physician burden and enhance patient education. **Methods**: An expert panel compiled thirteen frequently asked questions about RFA and cryoanalgesia, which were subsequently submitted to ChatGPT-4.0. The AI-generated responses were evaluated by 41 participants, including pain physicians, healthcare professionals, and non-healthcare individuals. The Likert scale was used for evaluating the responses, focusing on reliability, accuracy, and comprehensibility using predefined acceptability thresholds. **Results**: Across all 13 questions, ChatGPT’s responses demonstrated high overall reliability, with a mean score of 4.9 ± 0.7. The mean accuracy score was 2.6 ± 0.3, suggesting alignment with evidence-based standards. Comprehensibility was rated at 2.7 ± 0.2 on average. Pre-procedural questions achieved the highest scores, while post-procedural questions posed more challenges for ChatGPT. **Conclusions:** ChatGPT demonstrated potential as an adjunct tool for patient education on RFA and cryoanalgesia. Improvements in procedural specificity and medical accuracy are needed before routine clinical implementation.

## 1. Introduction

Radiofrequency ablation (RFA) and cryoanalgesia (also known as cryoneurolysis) are established interventional procedures in chronic pain management. RFA utilizes controlled heat administered via specialized probes to create a lesion in the targeted nerves, thereby interrupting pain signaling [1,2,3,4,5,6,7]. Cryoanalgesia, on the other hand, relies on the application of extreme cold to produce a reversible nerve conduction block, harnessing subzero temperatures to reduce nerve excitability [8]. Both techniques are minimally invasive modalities employed when patients who have failed and exhausted conservative medical management and have had positive diagnostic blocks, confirming the originator of pain [5,9,10]. As with most other chronic pain interventions, questions abound regarding RFA and cryoanalgesia by patients such as indications, feasibility, long-term effectiveness, and complications [7,11,12,13,14].

A key challenge in discussing these modalities with patients is presenting complex clinical information in a manner that is not only specific to the patient’s background, cultural, and educational background but must also foster trust in the treating physician and enhance informed decision-making by the patient. A possible solution to both the time constraints and tailored explanations is generative artificial intelligence (AI). Evidence continues to grow supporting the use of chatbots, such as ChatGPT, as possible adjuncts for providing accurate and comprehensible patient education by simplifying explanations, reducing the time needed to answer any concerns or questions, and clarifying procedural details [14,15,16,17,18].

However, research is sparse on the use of AI for patient education for RFA and cryoanalgesia. Our previous investigations on spinal cord stimulation (SCS) and opioid therapy suggest that although ChatGPT provides reliable, user-friendly responses on basic aspects, it may omit recent clinical developments and important details essential for effective patient education on said interventions [14,19]. Consequently, further investigation is necessary to determine whether ChatGPT can effectively communicate the nuances of RFA and cryoanalgesia. This study evaluated the reliability, accuracy, and comprehensibility of ChatGPT’s responses to commonly asked questions regarding RFA and cryoanalgesia. By replicating established assessment criteria from prior investigations [14,19], we aimed to identify areas of strength and improvement in AI-powered patient education for the aforementioned interventions. These procedures are frequently discussed during outpatient consultations, and patients often express concerns regarding indications, risks, expected benefits, and alternatives. In this context, large language models (LLMs), such as ChatGPT, may serve as accessible tools to enhance patient understanding and bridge communication gaps, especially when addressing common but technically nuanced topics like RFA and cryoanalgesia.

## 2. Materials and Methods

A previously validated protocol for assessing ChatGPT’s performance in patient education related to SCS and long-term opioid therapy was used for this study [14,19].

The protocol comprised three main steps: (i) identification of the most frequently asked patient questions through expert consensus, (ii) standardized submission of these questions to ChatGPT-4.0, and (iii) structured evaluation of the AI-generated responses by independent raters. This ensured reproducibility and comparability with previous investigations.

An expert panel composed of the study authors (GLB, CLR, MC, FPD, SN, ES, MM, EP, AT) compiled a list of 13 frequently asked questions regarding RFA and cryoanalgesia (Table 1). The selection process involved an initial open discussion based on each author’s clinical experience, followed by iterative refinement and ranking. The final 13 questions were selected by consensus as the most representative and frequently encountered in routine clinical practice [14,19].

The question selection process combined direct clinical experience, a focused review of existing patient education materials, and current clinical guidelines, to ensure that each question addressed distinct domains such as indications, mechanisms of action, procedural steps, clinical outcomes, risks, and post-procedural care. The finalized questions were categorized into three procedural domains: pre-procedural, intra-procedural, and post-procedural. To improve reproducibility, all prompts were submitted in a single session using a consistent browser and network environment. No follow-up or clarifying inputs were used to simulate a real-world patient interaction.

For instance, when asked *“What are radiofrequency ablation (RFA) and cryoanalgesia, and how do they help manage chronic pain?”*, ChatGPT generated a clear and structured explanation, which was generally rated highly across all domains. In contrast, for the question *“What should I do if I experience post-procedure discomfort, numbness, or any complications?”*, the response was limited to generic reassurance without highlighting specific red flags such as infection or motor weakness. This limitation reduced its accuracy rating.

The complete list of prompts and full, unedited responses are included in the Supplementary Materials.

All reviewers evaluated the responses independently and were blinded to each other’s assessments. Data were collected using standardized scoring forms, and inter-rater reliability was calculated using Cohen’s kappa coefficient;

All questions were submitted to the ChatGPT-4.0 model (OpenAI, San Francisco, CA, USA) on March 2025, using the standardized prompt: “If you were a pain physician, how would you answer a patient asking…?” The AI-generated responses were copied verbatim, without any editorial modifications.

A panel consisting of 41 participants—including pain physicians (*n* = 23), other healthcare professionals (*n* = 10), and non-healthcare individuals (*n* = 8)—independently rated the AI-generated responses. Participants were selected to provide a broad range of clinical and lay perspectives relevant to patient education.

**Figure 1:**
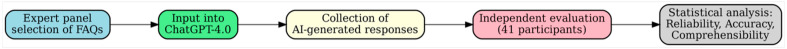
Flow chart of the study protocol. The process included five main steps: (1) selection of the most frequently asked patient questions on radiofrequency ablation (RFA) and cryoanalgesia by an expert panel; (2) standardized submission of these questions to ChatGPT-4.0; (3) collection of the AI-generated responses; (4) independent evaluation by 41 participants (pain physicians, healthcare professionals, and lay individuals); and (5) statistical analysis of reliability, accuracy, and comprehensibility.

To facilitate reader understanding, a flow chart summarizing the overall study design—from question generation to final analysis—has been included (Figure 1).

The evaluation was based on three domains: reliability, accuracy, and comprehensibility. Reliability was scored on a six-point Likert scale, assessing consistency, coherence, and trustworthiness of the response, with higher scores indicating closer alignment with recognized clinical guidelines. Accuracy was rated on a three-point Likert scale, measuring agreement with evidence-based information and current standards, where precise and comprehensive responses received the highest scores. Comprehensibility was evaluated on a three-point Likert scale, reflecting the clarity and ease of understanding from a patient’s perspective, with the highest score awarded to responses that were easy to follow and used minimal jargon. The previously used, predefined acceptability thresholds were set at reliability ≥ 4, accuracy ≥ 2, and comprehensibility ≥ 3 [14]. The use of a 6-point Likert scale for Reliability was intended to provide greater differentiation in perceived trustworthiness and coherence, while Accuracy and Comprehensibility were evaluated using 3-point scales to simplify judgments, especially for non-clinical participants. The acceptability threshold for Accuracy (≥2) corresponds to responses aligned with evidence-based information, whereas Comprehensibility required the highest possible score (3) to ensure optimal clarity and minimal jargon in patient-facing communication. These thresholds were consistent with our prior investigations [14,19].

**Figure 2:**
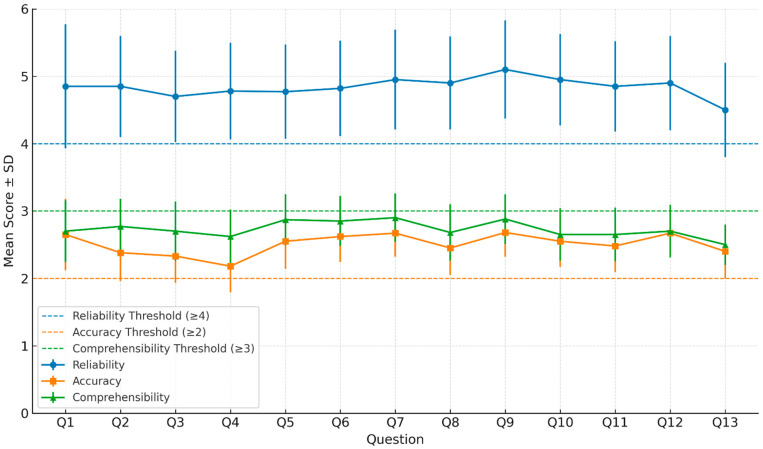
**Trends in Evaluator Scores Across Questions.** Line plot illustrating the mean reliability, accuracy, and comprehensibility scores (±standard deviation) for each of the 13 patient questions (Q1–Q13) regarding radiofrequency ablation and cryoanalgesia. Horizontal dashed lines indicate the predefined acceptability thresholds for each metric: reliability ≥ 4, accuracy ≥ 2, and comprehensibility ≥ 3. Error bars represent the standard deviation for each item, highlighting consistency and variability across evaluator ratings.

Descriptive statistics, including mean and standard deviation, were calculated for each question and each procedural domain using IBM SPSS Statistics v26. Scores were analyzed for patterns and trends, and normality was assessed through probability plots to confirm the appropriateness of the descriptive summaries. The study involved no direct collection of patient data, and institutional confirmation was obtained, indicating that formal review by the local Ethics Committee/Institutional Board Review was not required. During the preparation of this manuscript, the author(s) used ChatGPT 5.0 for the purposes of assisting in generating Figure 1 and Figure 2. The authors have reviewed and edited the output and take full responsibility for the content of this publication.

## 3. Results

Across all 13 questions, ChatGPT’s responses demonstrated high overall reliability, with a mean score of 4.9 ± 0.7 (Table 1 and Supplementary Table S1). Individual reliability ratings ranged from 4.7 to 5.1, indicating a consistent level of trustworthiness and coherence in the generated content. The mean accuracy score was 2.6 ± 0.3, with a range of 2.4 to 2.8, suggesting that the majority of responses were aligned with evidence-based standards, although some were noted as lacking in detail or depth. Comprehensibility was rated at 2.7 ± 0.2 on average, with values ranging between 2.5 and 2.9, reflecting generally clear and accessible language for patient-facing communication.

The total average ± SD across all questions was 4.85 ± 0.18 for reliability, 2.51 ± 0.16 for accuracy, and 2.69 ± 0.12 for comprehensibility (Supplementary Table S1).

When analyzing responses by procedural domain, pre-procedural questions (1–4) achieved the highest scores across all categories, with a reliability mean of 5.0 ± 0.6 and accuracy of 2.7 ± 0.2 (Figure 2 and Supplementary Table S1). These questions typically addressed definitions, indications, and candidate selection—topics where ChatGPT performed particularly well. Intra-procedural questions (5–9) had a high mean reliability of 4.9 ± 0.7 and accuracy of 2.6 ± 0.3. Post-procedural questions (10–13) yielded slightly lower mean scores, accuracy of 2.5 ± 0.4.

Topics such as post-procedure expectations, management of complications, and treatment durability posed more challenges for the AI model. For instance, the introductory question “What are RFA and cryoanalgesia?” (1) received one of the highest overall ratings, with mean scores of 4.85 ± 0.92 for reliability, 2.65 ± 0.53 for accuracy, and 2.70 ± 0.46 for comprehensibility. Conversely, the question addressing post-procedure complications (13), “What should I do if I experience post-procedure discomfort, numbness, or any complications?”, demonstrated a modest decline in performance, with mean scores of 4.5 ± 0.7 for reliability, 2.4 ± 0.4 for accuracy, and 2.5 ± 0.3 for comprehensibility. Overall, 93% of the evaluated responses met or exceeded the predefined acceptability thresholds in at least two out of three categories, while 75% of responses satisfied all three criteria simultaneously. A breakdown of evaluator composition is presented in Supplementary Table S2, to contextualize the diversity of perspectives in the rating process.

## 4. Discussion

Consistent with prior research on AI-driven education for SCS [14] and opioid therapy [19], ChatGPT demonstrated accurate and comprehensible information regarding RFA and cryoanalgesia. Similar investigations in other interventional pain contexts have reported comparable trends, with high reliability and clarity but variable accuracy, particularly for complex or post-procedural topics. In some hospital-based pilot programs, AI chatbots have been tested as supplementary tools for patient education, often under direct physician oversight. These implementations aimed to provide standardized, accessible information, streamline patient–provider communication, and reduce consultation time without replacing direct clinical interaction. Such real-world experiences support our findings that, while generative AI can effectively assist in delivering baseline educational content, its role must remain complementary to physician-led discussions, particularly for nuanced or safety-critical aspects of care.

In our analysis, ChatGPT did not significantly reduce physician workload in terms of consultation time, but it demonstrated potential as a complementary tool for patient education, particularly in clarifying pre-procedural aspects. Its contribution is therefore more qualitative (support in communication) rather than quantitative (time-saving).

Reliability remained high overall, suggesting that the risk of hallucinations (“AI hallucinations” refers to instances where the AI generates factually incorrect or fabricated information presented as if it were true, despite lacking any basis in verified sources) or unintentional fabrication of misleading information was minimal for the questions assessed. Nonetheless, the possibility of AI-generated hallucinations cannot be entirely excluded and may not have been detected within the 13 questions evaluated [20].

However, the content at times lacked up-to-date or specific details, mirroring known limitations when AI models rely on training data that may not reflect the latest medical literature [21,22,23]. To address these limitations, new AI platforms such as OpenEvidence—which integrates real-time access to PubMed and is developed in collaboration with leading scientific publishers—may offer improved accuracy by grounding responses in current and verifiable sources. Continuous monitoring and refinement of AI algorithms should be implemented to mitigate these errors, and it should be further emphasized that when using chatbots, the information should be considered purely as information and not used for diagnostic purposes with information provided verified by a physician [22,24,25].

This limitation was particularly evident in responses to post-procedural questions. For example, when asked the question (13), “What should I do if I experience post-procedure discomfort, numbness, or any complications?”, ChatGPT responded with broad reassurance and a generic suggestion to contact a physician, but failed to highlight red flags such as infection, motor weakness, or persistent sensory changes that warrant prompt medical attention. Similarly, in response to the question (12) “How long do the effects last and is repeat treatment necessary if my pain returns?”, ChatGPT offered only vague timelines without referencing differences in efficacy duration between RFA and cryoanalgesia, or the influence of individual patient factors. These gaps may limit the usefulness of AI-generated content based on an untrained model for setting realistic expectations and guiding timely follow-up. Similar patterns were observed in our prior assessments of ChatGPT’s performance in other interventional pain contexts. Future directions should include fine-tuning generative AI with domain-specific training sets, integration with updated clinical guidelines, and culturally adapted outputs. These improvements should also address critical gaps in post-procedural guidance and complications, as identified in our results. Moreover, multidisciplinary stakeholder feedback, including that from patient advocacy groups, could enhance the contextual relevance and trustworthiness of AI-generated responses.

Real-world implementation studies involving patient-reported feedback will be essential to assess practical usability, particularly in outpatient pain clinics.

In studies evaluating spinal cord stimulation (SCS) and long-term opioid therapy (LTOT), ChatGPT responses to general or introductory patient questions consistently scored higher than those addressing post-treatment care or technical nuances. This trend appears to persist in the context of RFA and cryoanalgesia, further highlighting the model’s difficulty with more complex clinical scenarios and the need for refinement when used in patient-facing applications [12,14,19].

From a clinical standpoint, both RFA and cryoanalgesia require patient-specific considerations such as treatment frequency, lesion parameters, comorbidities, and risk factors that are difficult for a generalized LLM to capture [8,14,15]. The omission of procedural nuances can influence patient expectations and shared decision-making. Therefore, while ChatGPT can serve as a helpful supplement, it should not replace direct discussions between patients and specialized pain physicians at the moment. To ensure patient-centered implementation, future models must be validated through real-world studies incorporating patient feedback, usability testing, and comparative effectiveness across diverse clinical settings. Moreover, implementing domain-specific refinement via curated training sets focusing on RFA and cryoanalgesia guidelines could significantly improve ChatGPT’s accuracy for more technical questions. Future directions should include fine-tuning generative AI with domain-specific training sets, integration with updated clinical guidelines, and culturally adapted outputs. Real-world implementation studies involving patient-reported feedback will be essential to assess practical usability, particularly in outpatient pain clinics.

Finally, although the evaluation included non-clinical participants, it did not involve patients directly. Future studies should aim to validate comprehensibility and clinical utility from a patient perspective, possibly using mixed-method approaches.

Our study included only one LLM (ChatGPT-4.0) without direct comparisons to other generative AI platforms limiting the generalizability. Moreover, though we included non-healthcare professionals, we did not obtain direct feedback from chronic pain patients undergoing RFA and cryoanalgesia, further restricting insights into real-world patient usage. Future research may incorporate multiple AI platforms and patient-centered evaluations.

## 5. Conclusions

ChatGPT demonstrated promising potential as an adjunct tool for patient education on RFA and cryoanalgesia. While its reliability and clarity are strong, improvements in procedural specificity and medical accuracy are needed before routine clinical implementation. Although future developments of general chatbots should focus on domain-specific model refinement, other chatbots, such as OpenEvidence, have been released, featuring integration with Pubmed and developed in collaboration with leading journals. Further studies could determine if the aforementioned chatbot would have improved results with procedural specificity and medical accuracy given that it pulls real-time data from Pubmed. In this study, ChatGPT did not replace or shorten consultations, but it proved helpful in supporting patient education by delivering reliable and comprehensible baseline information.

## Figures and Tables

**Table 1:** Frequently asked patient questions regarding radiofrequency ablation and cryoanalgesia. The following 13 questions were selected by majority consensus of the expert panel (comprising the study authors) based on their clinical experience and commonly encountered patient concerns in daily practice. The questions were designed to address key domains such as indications, mechanisms of action, procedural steps, expected outcomes, and potential risks, and were categorized into three procedural phases: pre-procedural, intra-procedural, and post-procedural.

Procedural Domains	Questions
**Pre-**	**1.** What are radiofrequency ablation (RFA) and cryoanalgesia, and how do they help manage chronic pain? **2.** Which pain conditions are most commonly treated with RFA or cryoanalgesia? **3.** Am I a suitable candidate for these procedures? How do I determine which one is more appropriate for my condition? **4.** Are there any risks or contraindications I should be aware of before proceeding?
**Intra-**	**5.** How is the procedure performed, and will I be awake or sedated during RFA or cryoanalgesia? **6.** What does it feel like when the nerve is heated or frozen, and is it painful? **7.** How long does each procedure typically take, and do they differ in duration? **8.** How do you ensure the correct nerve or location is targeted during the procedure? **9.** Can I receive RFA and cryoanalgesia at the same time or in different sessions if needed?
**Post-**	**10.** What kind of relief can I expect afterward, and how quickly will I notice improvements in my pain? **11.** Are there any activity restrictions following RFA or cryoanalgesia, and how long should I limit certain movements? **12.** How long do the effects last, and is repeat treatment necessary if my pain returns? **13.** What should I do if I experience post-procedure discomfort, numbness, or any complications?

## Data Availability

The data presented in this study are available from the corresponding author upon reasonable request.

## Supplementary Materials

The supporting information can be downloaded at https://www.mdpi.com/article/10.3390/jcm14196814/s1. Supplementary Table S1. Mean Evaluator Scores per Question; Supplementary Table S2. Evaluator Panel Composition; Supplementary Table S3. Distribution of reliability scores (6-point Likert scale) for each of the 13 patient questions regarding radiofrequency ablation and cryoanalgesia, as evaluated by the panel (*n* = 41); Supplementary Table S4. Distribution of Accuracy Scores (3-point Likert scale); Supplementary Table S5. Distribution of Comprehensibility Scores (3-point Likert scale).
