# Correction: Lo Bianco et al. Assessing Artificial Intelligence-Powered Responses to Common Patient Questions on Radiofrequency Ablation and Cryoanalgesia for Chronic Pain. *J. Clin. Med.* 2025, *14*, 6814

**DOI:** 10.3390/jcm14228157

**Published:** 2025-11-18

**Authors:** Giuliano Lo Bianco, Marco Cascella, Silvia Natoli, Francesco Paolo D’Angelo, Emanuele Sinagra, Maurizio Marchesini, Emanuele Piraccini, Andrea Tinnirello, Felice Occhigrossi, Cesare Gregoretti, Christopher L. Robinson

**Affiliations:** 1 Department of Anaesthesia and Intensive Care, Fondazione Istituto “G. Giglio” Cefalù, 90015 Palermo, Italy; 2 Anesthesia and Pain Medicine, Department of Medicine, Surgery and Dentistry “Scuola Medica Salernitana”, University of Salerno, 84081 Baronissi, Italy; 3 Department of Clinical-Surgical, Diagnostic and Pediatric Sciences, University of Pavia, 27100 Pavia, Italy; 4 Pain Unit, Fondazione IRCCS Policlinico San Matteo, 27100 Pavia, Italy; 5 Department of Anaesthesia, Intensive Care and Emergency, University Hospital Policlinico Paolo Giaccone, 90127 Palermo, Italy; 6 Gastroenterology and Endoscopy Unit, Fondazione Istituto San Raffaele Giglio, 90015 Cefalù, Italy; 7 Department of Anesthesia and Pain Medicine, Mater Olbia Hospital, 07026 Olbia, Italy; marchesinidoc@gmail.com; 8 Unit of Pain Management, Emergency Department, AUSL Bologna, 40033 Bologna, Italy; 9 Anesthesia and Pain Management Unit, ASST Franciacorta, Iseo Hospital, 25049 Brescia, Italy; 10 Pain Therapy Unit, San Giovanni-Addolorata Hospital, 00184 Rome, Italy; 11 Faculty of Medicine and Surgery, Saint Camillus International University of Health and Medical Sciences “UniCamillus”, 00131 Rome, Italy; 12 Division of Pain Medicine, Department of Anesthesiology and Critical Care, School of Medicine, Johns Hopkins University, Baltimore, MD 21205, USA

Additional Affiliations

In the original publication [1], there were two errors regarding the affiliations for Cesare Gregoretti. Instead to the affiliation Anesthesiology and Pain Department, Foundation G. Giglio Cefalù, 90015 Palermo, Italy, the updated affiliations should include the following: Department of Anaesthesia and Intensive Care, Fondazione Istituto “G. Giglio” Cefalù, 90015 Palermo, Italy, and Faculty of Medicine and Surgery, Saint Camillus International University of Health and Medical Sciences “UniCamillus”, 00131 Rome, Italy. The authors state that the scientific conclusions are unaffected. This correction was approved by the Academic Editor. The original publication has also been updated.
