# Supplementary material for: Assessing Artificial Intelligence-Powered Responses to Common Patient Questions on Radiofrequency Ablation and Cryoanalgesia for Chronic Pain
Source: J Clin Med. 2025 Sep 26;14(19):6814. doi: 10.3390/jcm14196814 (PMC12525085; doi:10.3390/jcm14196814)
Supplement: Supplementary file 1 [file jcm-14-06814-s001.zip › jcm-3813905-supplementary.pdf]

Supplementary Tables

Supplementary Table S1. Mean Evaluator Scores per Question

Mean scores (± standard deviation, SD) of reliability, accuracy, and comprehensibility for each of the thirteen frequently asked patient questions regarding radiofrequency ablation and cryoanalgesia, as rated by the expert panel.

Question	Reliability (Mean ± SD)	Accuracy (Mean ± SD)	Comprehensibility (Mean ± SD)
Q1	4.85 ± 0.92	2.65 ± 0.53	2.7 ± 0.46
Q2	4.85 ± 0.75	2.38 ± 0.42	2.77 ± 0.41
Q3	4.7 ± 0.68	2.33 ± 0.4	2.7 ± 0.44
Q4	4.78 ± 0.72	2.18 ± 0.39	2.62 ± 0.4
Q5	4.77 ± 0.7	2.55 ± 0.41	2.87 ± 0.38
Q6	4.82 ± 0.71	2.62 ± 0.38	2.85 ± 0.37
Q7	4.95 ± 0.74	2.67 ± 0.35	2.9 ± 0.36
Q8	4.9 ± 0.69	2.45 ± 0.4	2.68 ± 0.42
Q9	5.1 ± 0.73	2.68 ± 0.36	2.88 ± 0.37
Q10	4.95 ± 0.68	2.55 ± 0.38	2.65 ± 0.39
Q11	4.85 ± 0.67	2.48 ± 0.39	2.65 ± 0.4
Q12	4.9 ± 0.7	2.67 ± 0.36	2.7 ± 0.39
Q13	4.5 ± 0.7	2.4 ± 0.4	2.5 ± 0.3

Supplementary Table S2. Evaluator Panel Composition

This table summarizes the composition of the evaluator panel that assessed the AI-generated responses. A total of 41 participants were included, representing a range of perspectives relevant to patient education. The panel consisted of 23 pain physicians, 10 other healthcare professionals (e.g., nurses, physiotherapists), and 8 non-healthcare individuals. This distribution ensured a balanced evaluation across clinical and lay viewpoints, enhancing the generalizability of the findings.

**Evaluator Type**	**n**
**Pain physicians**	23
**Other healthcare providers**	10
**Non-healthcare individuals**	8
**Total**	41

Supplementary Table S3. Distribution of reliability scores (6-point Likert scale) for each of the 13 patient questions regarding radiofrequency ablation and cryoanalgesia, as evaluated by the panel (n = 41).

Question	Score 1	Score 2	Score 3	Score 4	Score 5	Score 6
**Q1**	2	22	16	0	0	0
**Q2**	1	12	27	0	0	0
**Q3**	3	21	14	2	0	0
**Q4**	0	9	31	0	0	0
**Q5**	3	21	16	0	0	0
**Q6**	0	12	28	0	0	0
**Q7**	1	16	23	0	0	0
**Q8**	1	7	28	0	2	0
**Q9**	2	16	21	0	0	0
**Q10**	1	11	27	0	0	0
**Q11**	3	18	19	0	0	0
**Q12**	0	14	26	0	0	0
**Q13**	3	16	21	0	0	0

Supplementary Table S4. Distribution of accuracy scores (3-point Likert scale) for each of the 13 patient questions regarding radiofrequency ablation and cryoanalgesia, as evaluated by the panel (n = 41).

Question	Score 1	Score 2	Score 3
**Q1**	3	27	10
**Q2**	3	27	10
**Q3**	3	27	10
**Q4**	3	27	10
**Q5**	1	21	17
**Q6**	1	21	17
**Q7**	1	21	17
**Q8**	1	21	17
**Q9**	1	21	17
**Q10**	0	20	20
**Q11**	0	20	20
**Q12**	0	20	20
**Q13**	0	20	20

Supplementary Table S5. Distribution of comprehensibility scores (3-point Likert scale) for each of the 13 patient questions regarding radiofrequency ablation and cryoanalgesia, as evaluated by the panel (n = 41)

Question	Score 1	Score 2	Score 3
**Q1**	1	13	26
**Q2**	1	13	26
**Q3**	1	13	26
**Q4**	1	13	26
**Q5**	0	6	34
**Q6**	0	6	34
**Q7**	0	6	34
**Q8**	0	6	34
**Q9**	0	6	34
**Q10**	1	11	27
**Q11**	1	11	27
**Q12**	1	11	27
**Q13**	2	8	27
